# Plantar Purpura as the Initial Presentation of Viridians Streptococcal Shock Syndrome Secondary to* Streptococcus gordonii* Bacteremia

**DOI:** 10.1155/2016/9463895

**Published:** 2016-04-17

**Authors:** Chen-Yi Liao, Kuan-Jen Su, Cheng-Hui Lin, Shu-Fang Huang, Hsien-Kuo Chin, Chin-Wen Chang, Wu-Hsien Kuo, Ren-Jy Ben, Yen-Cheng Yeh

**Affiliations:** ^1^Department of Internal Medicine, Kaohsiung Armed Forces General Hospital, No. 2 Zhongzheng 1st Road, Lingya District, Kaohsiung 802, Taiwan; ^2^Department of Cardiovascular Surgery, Kaohsiung Armed Forces General Hospital, No. 2 Zhongzheng 1st Road, Lingya District, Kaohsiung 802, Taiwan

## Abstract

Viridians streptococcal shock syndrome is a subtype of toxic shock syndrome. Frequently, the diagnosis is missed initially because the clinical features are nonspecific. However, it is a rapidly progressive disease, manifested by hypotension, rash, palmar desquamation, and acute respiratory distress syndrome within a short period. The disease course is generally fulminant and rarely presents initially as a purpura over the plantar region. We present a case of a 54-year-old female hospital worker diagnosed with viridians streptococcal shock syndrome caused by* Streptococcus gordonii*. Despite aggressive antibiotic treatment, fluid hydration, and use of inotropes and extracorporeal membrane oxygenation, the patient succumbed to the disease. Early diagnosis of the potentially fatal disease followed by a prompt antibiotic regimen and appropriate use of steroids are cornerstones in the management of this disease to reduce the risk of high morbidity and mortality.

## 1. Introduction

Toxic shock syndrome (TSS) is an acute, toxin-mediated, and life-threatening illness, generally precipitated by infection with either* Staphylococcus aureus* or group A beta-hemolytic* Streptococcus* (GAS;* Streptococcus pyogenes*). It is characterized by high fever, rash, hypotension, multiorgan failure (involving at least three or more organ systems), and desquamation, typically of the palms and soles, 1-2 weeks after the onset of acute illness. Recently, a growing number of cases of another toxic shock-like syndrome manifested by alpha-hemolytic (viridians) streptococci named “viridians streptococcal shock syndrome” (VSSS) have been reported. The diagnosis usually requires microbiological evidence of viridians group streptococci (VGS) from blood culture and clinical manifestation characterized by hypotension, rash, palmar desquamation, and adult respiratory distress syndrome. The typical dermatologic presentation of VSSS generally starts with a rash and desquamation of the palms or soles, which occur 8–14 days after the onset of infection, presenting with a maculopapular appearance centrifugally spreading from the trunk to the face and extremities. VSSS may occur early or within 2-3 days after presentation and progress to respiratory failure within 48 h.

The viridians group streptococci (VGS) are a group of catalase-negative, Gram-positive cocci composed of heterogeneous groups of organisms with complex and controversial taxonomy. They are subclassified into six major groups, including the* S*.* mutans* group,* S*.* salivarius* group,* S*.* anginosus* group,* S*.* mitis* group,* S*.* sanguinis* group, and* S*.* bovis* group [1, 2]. Some taxonomists have lumped the* S. sanguinis* group in with the* S. mitis* group based on 16S rRNA gene sequence analysis, but* S. sanguinis* group organisms exhibit divergent phenotypic characteristics. The* S*.* sanguinis* group includes* S*.* sanguinis*,* S*.* parasanguinis*, and* S*.* gordonii* [1]. The VGS are pathogens with low virulence that are generally commensal in the oral cavity, upper airways, the gastrointestinal tract, and the female genital tract [3–5]. These organisms may invade sterile body sites, causing a series of infectious diseases such as orbital cellulitis [6], endophthalmitis [7], pneumonia [8], vertebral osteomyelitis [9], and bacteremia [10] and may even lead to life-threatening diseases such as endocarditis [2], meningitis [5], and toxic shock-like syndrome [4]. Although VGS disease may occur in healthy hosts, it most commonly manifests in those with underlying conditions, including immunosuppression or cardiac abnormalities [2].

The clinical presentation of* S*.* gordonii* infection may present as subacute bacterial endocarditis [11], septic arthritis [12], spontaneous bacterial peritonitis [13], and multiple subcutaneous abscesses [14].

To our knowledge,* S. gordonii* has never been reported as indolent prodrome with plantar pupular and rapid progressive VSSS. We present a case of a 54-year-old female with* S*.* gordonii*-related VSSS with a fatal outcome.

## 2. Case Presentation

**Figure 1:**
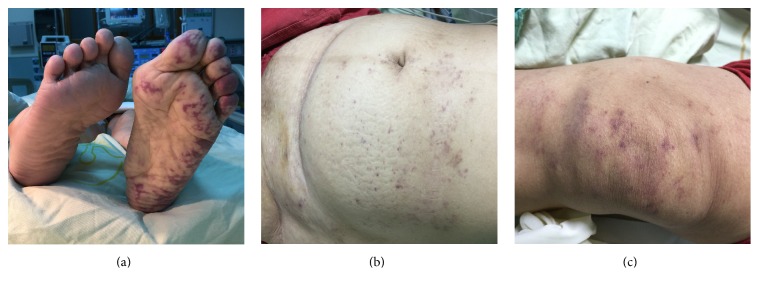
(a) A tattoo-like purpuric rash over the left plantar region was observed. (b) Petechiae on the lower abdomen region. (c) Petechiae on the left knee region with no obvious pitting edema on the left lower limb or knee joint were noted.

A 54-year-old female hospital worker with a history of gout and valvular heart disease with moderate mitral regurgitation presented to the emergency department with a 1-month history of intermittent left foot pain and numbness extending from the knee to the calf region. She then developed acute upper respiratory tract infection, presenting with sore throat, rhinorrhea, and productive cough 11 days prior to admission. Subsequently, she had visited the orthopedic and neurology outpatient department 7 days prior to admission because of progressive numbness in the left calf region and limited range of motion when lifting her left foot, with symptoms persisting despite meticulous examination and treatment. She experienced poor appetite, general malaise, progressive arthralgia, and myalgia, particularly in the left foot. She had also called the dermatology outpatient department 3 days prior to admission because of progressive petechiae and purpura formation on her left plantar region. The patient used anxiolytic agents and sleeping pills. She denied the use of illicit drugs. She had no recent history of traveling. Besides, she had dental extraction 2 years ago with proper dentition. Physical examination revealed normal body temperature (35.9 degrees Celsius), rapid heart rate (110 beats/min), and low blood pressure (71/46 mmHg). The facial skin was intact, and there was no erythema, rash, or blisters. Examination of the oral cavity revealed an edematous tongue, pharynx, and buccal mucosa with no appearance of mucositis. Neck stiffness and localizing neurological signs were not observed, and her Glasgow coma score was 14. No redness of eye, vision changes, conjunctival suffusion, or Roth spots were noticed in eye examination. Her left lower limb examination was unremarkable with respect to soft tissue or skeletal injury. No desquamation of palms, Osler's node, or Janeway lesion was observed. Purpura on the left plantar region in the absence of blisters was observed 3 days prior to admission (Figure 1(a)), and petechiae on the lower abdomen (Figure 1(b)) and the left knee region (Figure 1(c)) were noticed 5 days prior to admission. The arms, posterior back, and right leg did not have any skin rash. No obvious pitting edema over the left lower limb and knee joint was noted (Figure 1(c)). No joint effusion or bone pains were noticed. Palpation of the soft tissues was nontender, with no signs of grimacing or withdrawal, and the consistency was soft with no signs of compartment syndrome. Active movements at the hip, knee, and ankle joints were within the normal range and not associated with pain. All peripheral pulses were normal with satisfactory capillary circulation. No dysesthesia or loss of proprioception was noticed in the lower extremity. Laboratory investigation revealed mild anemia with a hemoglobin of 10^9^ g/L (normal range, 115–155), platelet count of 175 × 10^9^/L (normal range, 150–400), leukocytosis with a white blood cell count of 25.0 × 10^9^/L (normal range, 4.0–11.0), and a marked elevation of neutrophils (93.8%). Total bilirubin was 4.27 *μ*mol/L (normal range, 5.1–17.1), alanine aminotransferase was 0.78 *μ*kat/L (normal range, 0.11–0.86), aspartate aminotransferase was 0.43 *μ*kat/L (normal range, 0.21–0.65), creatinine was 106.7 *μ*mol/L (normal range, 53.3–99.1), C-reactive protein was 114.8 nmol/L (normal, <9.5), and albumin was 15 g/L (normal range, 35–57). Urine examination showed mild proteinuria, leukocyturia, and erythrocyturia.

**Figure 2:**
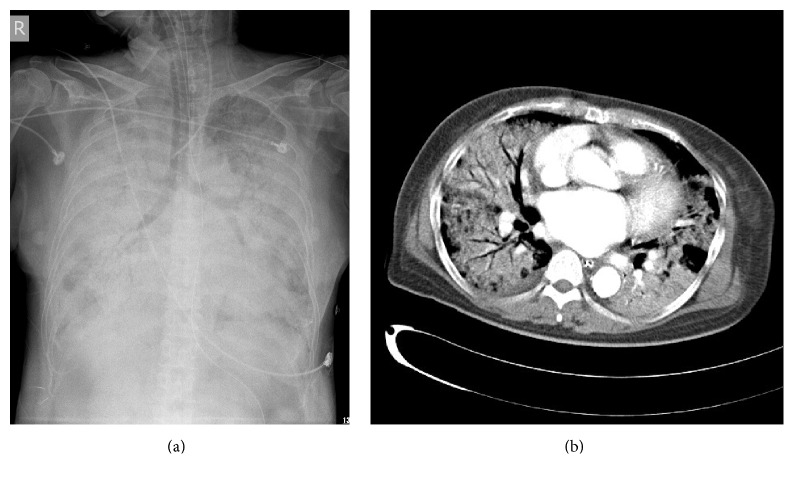
(a) A second chest X-ray showed bilateral consolidation with a snowflake pattern (8 h upon admission). (b) Chest computed tomography of the lungs demonstrated diffuse bilateral alveolar and interstitial infiltrations with a presentation compatible with acute respiratory distress syndrome (ARDS).

Initial chest X-ray revealed an increased lung marking over the right lower lung. A provisional diagnosis of cellulitis was made, and, upon microbiological advice, treatment was commenced with oxacillin (2 g) administered intravenously every 4 h as an empirical regimen. The patient started to have hemoptysis, developed hypotension, and progressive respiratory distress within 2 hours after admission. She was transferred to the intensive care unit (ICU) in acute respiratory failure (arterial blood gas analysis revealed a pH of 7.233, a pCO_2_ of 63.5 mmHg, and a pO_2_ of 44.4 mmHg with an FiO_2_ of 40%) and was intubated and placed on a ventilator. A second chest X-ray showed bilateral consolidation with a snowflake pattern (Figure 2(a)). Subsequently, the patient was placed on a lung-protective strategy and shifted to broad-spectrum antibiotics, including vancomycin (1.0 g) administered intravenously every 12 h, ceftriaxone (2.0 g) administered intravenously every day, and ciprofloxacin (400 mg) administered intravenously every 12 h. Chest computed tomography demonstrated diffuse alveolar and bilateral lung interstitial infiltrations with a presentation compatible with acute respiratory distress syndrome (ARDS; Figure 2(b)). While arriving at the intensive care unit, she developed unstable atrial fibrillation and returned to sinus rhythm after administration of antiarrhythmic agents (amiodarone) and three series of successful cardioversions. Transthoracic echocardiography showed mild to moderate mitral regurgitation and no evidence of vegetation. Because of the occurrence of refractory ARDS with severe hypercapnia and hypoxemia, the patient received 3 h of venovenous extracorporeal membrane oxygenation (ECMO) 1 day after admission (arterial blood gas analysis revealed a pH of 7.523, a pCO_2_ of 24.8 mmHg, and a pO_2_ of 50.3 mmHg with an FiO_2_ of 100%). Despite the administration of inotropic agents and high-dose norepinephrine, the patient succumbed to the disease after progressive bradycardia and fulminant shock.

Because of episodes of hemoptysis and a clinical picture of probable alveolar hemorrhage, we requested a complete infectious survey for possible etiology, including sputum culture, tuberculosis/acid fast stain, serology with enzyme-linked immunosorbent assay (ELISA) for Hantavirus, microscopic agglutination test for leptospirosis, and ELISA for human immunodeficiency virus (HIV), which eventually yielded unremarkable results. Autopsy was unavailable because of denial from the family; thus no objective evidence of bacterial endocarditis from heart tissue was obtained. The growth of* S. gordonii* was observed by 2 sets of blood cultures collected 4 hours apart using Becton Dickinson Diagnostic Instrument Systems (BACTEC). The blood culture was done on admission and showed positivity 3 days later. The bacterial isolates showed susceptibility to clindamycin, ceftriaxone, vancomycin, erythromycin, and levofloxacin.

## 3. Discussion

A definitive diagnosis was not made prior to death because of the rapid disease progression after admission following an 11-day mild prodrome. The possible reasons for this delay include the vague history and nonspecific clinical features, the absence of a clinically detectable septic focus in a previously healthy patient, and the time required for organisms to be cultured in the laboratory. The blood culture in our patient yielded* S*.* gordonii*, which belongs to the* S*.* sanguinis* group, and is one of the VGS, a genetically heterogeneous group of bacteria predominating in the human oropharynx [15]. The risk factors associated with VGS that have been identified are oral mucositis [16], profound neutropenia, high-dose chemotherapy like cytosine arabinoside, malignancy, particularly in pediatric patients with leukemia [17] and neutropenic cancer [10] and patients undergoing stem-cell transplants [18], antimicrobial prophylaxis with trimethoprim-sulfamethoxazole or fluoroquinolone, and the use of antacids, histamine type 2 receptor antagonists, or proton pump inhibitors [19, 20]. Our patient did not have oral symptoms and showed no evidence of mucositis. Mucositis is the most common route of entry for these organisms; however, in the study conducted by Gamis et al., nearly half of the cases of bacteremia (45%) occurred in patients without mucositis, suggesting that other mechanisms of entry are also important. In those that became bacteremic in the absence of mucositis, gastrointestinal toxicity was implicated as a potential risk factor [21].

The clinical course of VGS bacteremia is variable. Most cases present with minimal symptoms with complete recovery; others may present with a toxic shock-like syndrome known as VSSS characterized by hypotension, rash, palmar desquamation, and ARDS developing upon the onset of bacteremia in approximately 25% in the normal population [10] and in 13%–21% in children after bone marrow transplantation [18]. The mortality rate among patients with VGS bacteremia who develop complications is high: up to 80% in some case series and between 40% and 100% in children [18]. The current reported VGS species related VSSS include* S. mitis*,* S. oralis, and S. viridans* [20].* S. mitis* is the most common VGS species associated with VSSS compared to non-*S. mitis* organisms [2, 19]. According to a study by Shelburne et al., patients infected with* S. mitis* strains were more likely to have moderate or severe clinical disease (e.g., VSSS) than those infected with non-*S. mitis* strains [22]. Although S.* gordonii* is one of the* S. sanguinis* group strains, it has seldom been reported as being implicated in VSSS.

The blood culture of* S. gordonii* in our patient showed susceptibility to clindamycin, ceftriaxone, vancomycin, erythromycin, and levofloxacin. Despite antibiotic therapy with vancomycin, ceftriaxone, and ciprofloxacin (quinolone), our patient succumbed to the VSSS which may be due to delayed proper antibiotic use in face of the mild prodrome. Through selective pressure, antibiotics that target Gram-negative organisms, such as quinolones, may promote VGS expansion and increase the risk of a patient becoming bacteremic [23]. A study conducted by Han et al. showed that isolation of a levofloxacin-resistant organism was linked to prophylactic use of quinolones [19]. Yacoub et al. proposed that the administration of moderate dose corticosteroids with short administration duration may be beneficial for preventing the development of ARDS in patients with* S*.* mitis* bacteremia [20]. Yacoub et al. carried out a study among a group of VSSS patients and reported a 100% recovery from ARDS. Though the case number is small, the study results may give VSSS a novel direction for effective therapy [20]. Our patient did not receive corticosteroid therapy during the hospital course due to no definite evidence of initial corticosteroid use according to the ARDS therapy guidelines.

VGS endocarditis generally presents with an indolent course, involving prolonged low-grade fever and a variety of somatic complaints such as arthralgia, myalgia, weight loss, rigors, fatigue, weakness, and diaphoresis, which are symptoms mimicking the clinical course described in the present case. Infective endocarditis is a life-threatening disease caused by a bacterial infection of the endocardial surfaces of the heart. The oral pathogen* S*.* gordonii* is among the most common pathogens isolated from infective endocarditis patients.* S*.* gordonii* initiates dental plaque formation and endocarditis by entering into the blood stream, generally upon oral trauma [24].* S*.* gordonii* colonizes platelet-fibrin thrombi, blood-clotting agents, in damaged heart valves or the endocardium, leading to damage and dysfunction of the heart valves [25].

TEE (transesophageal echocardiogram) is unavailable in our case and transthoracic echocardiography showed mild to moderate mitral regurgitation and no evidence of vegetation. The relevant pathogen in our patient fulfilled one major criterion according to the modified Duke infective endocarditis criteria [26]. The past history of mitral regurgitation in our patient is a probable predisposing risk factor for the development of VGS endocarditis. However, the patient had no fever, vascular phenomena, or immunologic phenomena, which makes the current case fulfill only one minor criterion. The plantar lesion with the presentation of scattered purpura does not mimic the clinical presentation of typical Janeway lesion which is the common vascular phenomena of infective endocarditis manifested as irregular painless erythematous macules on the palm and soles. Thus, according to the modified Duke infective endocarditis criteria, infective endocarditis in our patient could be possible. We could not completely exclude this diagnosis because of the lack of TEE. In our patient, the indolent symptoms were indicative prodrome of endocarditis and 11 days later the patient developed VSSS.

## 4. Conclusion

The atypical presentation of* S*.* gordonii*-related VSSS in our case included a mild prodrome including myalgia, arthralgia, and purpura with dominant symptoms only on the left foot lasting for approximately 2 weeks, which is easily overlooked and misdiagnosed. The disease course rapidly progressed into shock and ARDS within only 1 day of hospitalization, despite aggressive treatment. This patient had many demanding care issues to be treated in a quickly moving scenario; however, early identification of this prodrome with timely antibiotic use and corticosteroids might be beneficial in cases with VSSS.
